# Loss of EPB41L3: a common molecular link in the tumorigenesis of neurofibromatosis types 1 and 2

**DOI:** 10.3389/fonc.2026.1632602

**Published:** 2026-05-20

**Authors:** Erxing Tao, Lin Zhou, Zhicheng Xu, Hui Ren, Junyu Yang, Yiming Chen, Qi Wang, Min Liu, Yanfei Zhang, Wei Liang, Siyi Xu, Chunlong Zhong, Zhigang Wang

**Affiliations:** 1 Department of Neurosurgery, The First Affiliated Hospital of Nanchang University, Nanchang, Jiangxi, China; 2 Department of Neurosurgery, Shanghai East Hospital, School of Medicine, Tongji University, Shanghai, China; 3 Department of Neurosurgery, Shanghai Tenth People's Hospital, Tongji University School of Medicine, Shanghai, China; 4 Department of Central Laboratory, Shanghai Tenth People's Hospital, Tongji University School of Medicine, Shanghai, China

**Keywords:** EPB41L3, LOH, mutation, neurofibromatosis type 1, neurofibromatosis type 2, proteomics

## Abstract

**Background:**

Neurofibromatosis type 1 (NF1) and Neurofibromatosis type 2 (NF2) are autosomal dominant disorders that originate from Schwann cells and are characterized by the development of benign and malignant tumors, respectively. This study investigated the genetic and proteomic profiles of tumors.

**Methods:**

Mutational analyses were performed on the neurofibromatosis type 1 and type 2 genes (*NF1* and *NF2*) in 15 NF1 and NF2 patients using Sanger sequencing. TMT-labeled spectrometry was employed to identify the proteomic differences between NF1 and NF2 samples and nerve controls. The loss of heterozygosity (LOH) of the candidate gene *EPB41L3* was analyzed. Protein expression in tumor tissues and cell cultures was evaluated using Western blotting and/or immunofluorescence techniques.

**Results:**

DNA sequencing revealed frequent mutations within the *NF1* and *NF2* genes in patients with neurofibromatosis type 1 and type 2, respectively. Compared with normal nerves, RNA sequencing revealed that a total of 159 proteins were significantly differentially expressed in patients with NF1, and 180 proteins were differentially expressed in patients with NF2. Among these, 11 proteins were commonly upregulated and 30 were commonly downregulated in both tumor types. Notably, *EPB41L3* was consistently found to be significantly downregulated in both tumors and was predominantly localized in the cytoplasm of HEI193 and NF1 cell cultures. Western blot (WB) analysis indicated that the downregulation in both tumor types was associated with the expression of neurofibromin and Merlin. Loss of heterozygosity (LOH) of *EPB41L3* was more prevalent in patients with NF1 (60%) than in patients with NF2 (20%). Furthermore, the overexpression of *EPB41L3* inhibited HSC proliferation and cell viability, and significantly upregulates the expression of Merlin and Neurofibromin, whereas *EPB41L3* knockdown significantly increased HSC proliferation and viability, and significantly downregulates the expression of Merlin and Neurofibromin.

**Conclusions:**

Our data provide an initial insight into the common molecular mechanisms underlying neurofibroma, suggesting that *EPB41L3* could serve as a potential therapeutic target for this tumor syndrome.

## 1 Introduction

Neurofibromatosis (NF) encompasses two distinct syndromes, both of which originate from the Schwann cells of the peripheral nerve sheath: neurofibromatosis type 1 (NF1) and neurofibromatosis type 2 (NF2) (1, 2). NF1 is a rare disease, with an estimated birth incidence of 1 in 2,500 to 3,000 individuals, whereas the birth incidence of NF2 is lower, with estimates ranging from 1 in 25,000 to 40,000 (1, 2). These two syndromes are autosomal dominant disorders caused by germline mutations in the *NF1* and *NF2* tumor suppressor genes, respectively (1–3). Mutations in tumor suppressor genes (*NF1* and *NF2*) lead to the formation of neurofibromas. Mutations in the *NF1* gene can give rise to various types of neurofibromas, including cutaneous (cNF), subcutaneous (scNF), and plexiform (pNF) forms, whereas bilateral schwannomas are the most characteristic lesions of *NF2* gene mutation, and are rarely seen in *NF1* (1, 4, 5). Although the two neoplastic syndromes are fundamentally different in terms of their pathogenic genes, they still have some pathophysiological similarities.

The *NF1* gene is located on chromosome 17q11.2, and most of its mutations result in the functional loss of neurofibromin (6). Neurofibromin is a GTPase-activating protein (GAP) that plays a crucial role in maintaining the inactive form of the RAS protein (7). The loss of neurofibromin leads to increased RAS activity, resulting in the deregulation of several signaling pathways, including the RAS–MAPK (Raf/MEK/ERK) and PI3K/AKT/mTOR pathways, which promote increased cell proliferation and cell division and ultimately contribute to tumorigenesis in neurocutaneous tissues. Neurofibromin is associated with cytoplasmic microtubules and polymerized microtubules within cells (8, 9). Some authors have reported that neurofibromin is involved in microtubule-mediated intracellular signal transduction pathways through interactions within the GAP-related domain (9).

The *NF2* gene encodes Merlin, which functions as a regulator of growth, motility, and motogenic signaling (10). Loss of Merlin/NF2 leads to the deregulation of multiple signaling pathways, including the RAS–RAF–MEK–ERK (MAPK) pathway, various membrane receptors, and members of the Rho GTPase family, which can contribute to tumorigenesis (11, 12). To some extent, the signaling pathways affected by NF2 and NF1 in tumorigenesis may overlap. The tumor suppression function of neurofibromin, encoded by the *NF1* gene, primarily involves the accelerated inactivation of RAS. In contrast, Merlin, encoded by *NF2*, coordinates multiple signaling pathways (13). Studies have demonstrated that Merlin interacts with Ras signaling components, including RAF1, through protein–protein interactions within multiprotein signaling complexes (13). The Ras binding domain (RBD) is present in RAF1 but not in Merlin, suggesting a regulatory role for.

Merlin in these pathways. NF2 is a component of kinesin-1-containing complexes, as identified through immunoprecipitation experiments using both NH2- and COOH-terminal anti-NF2 antibodies (14, 15). NF1 is also a component of large kinesin-1-containing complexes, indicating that fractions of NF2 and NF1 may have stable associations (15). Given the similarities in their molecular structures, NF1 and NF2 may share common mechanisms in pathogenesis, suggesting that understanding the interactions and functions of these proteins could provide insights into their roles in disease processes.

Overall, these two distinct genes may play similar roles in tumorigenesis. However, the precise mechanisms through which Merlin and neurofibromin inhibit these pathways remain elusive. LC–MS has emerged as an efficient high-throughput method for probing the underlying changes in tumorigenesis. In this context, we introduce these two tumor types to explore their common pathogenic targets. Furthermore, a systematic examination of the pathways disrupted by the loss of neurofibromin (encoded by *NF1*) and Merlin (encoded by *NF2*) function has facilitated therapeutic progress for patients with neurofibroma. Erythrocyte membrane protein band 4.1-like 3 (*EPB41L3*) is a human gene encoding a member of the 4.1 family of cytoskeletal proteins, which are involved in linking the plasma membrane to the underlying cytoskeleton (16). Studies have shown that the EPB41L3 protein attenuates cell migration and epithelial-to-mesenchymal transition (EMT), promotes apoptosis, and thereby suppresses tumor proliferation (17–19). This protein is a membrane–cytoskeleton linker with dual roles in tumor suppression (16, 20). Nevertheless, the precise mechanism through which EPB41L3 expression exerts its inhibitory effects in the context of neurofibromatosis remains unclear.

## 2 Materials and methods

### 2.1 Sample collection

In our study, we analyzed fifteen samples from patients with hereditary type 1 and type 2 neurofibromatosis obtained during surgical operations in our department. Clinical diagnosis of neurofibromatosis type 1 and type 2 was based on medical history, physical examination, characteristic MR imaging features, pathological diagnosis and operative findings. All the samples were subjected to histological examinations to confirm the diagnosis. The tumors were either preserved at −80 °C until DNA/protein extraction or collected immediately for primary culture after resection. Three great auricular nerves obtained during parotidectomy were included as nontumoral controls. Informed written consent was obtained from all patients who donated tissue. This study was approved by the Institutional Review Board of the Ethics Committee of The Shanghai East Hospital, which is affiliated with the School of Medicine, Shanghai Tong Ji University.

### 2.2 DNA extraction and sequencing

Sanger sequencing was conducted to detect mutations in *NF1* and *NF2*. DNA was extracted from the tumor samples using the TIANamp Genomic DNA Kit (Tiangen Biotech, Beijing, China). The whole coding sequence and exon–intron boundaries of the genes were amplified by polymerase chain reaction (PCR) using standard methods and underwent bidirectional sequencing. The sequence data were analyzed using Sequencer 4.9 software (Gene Codes Corporation, Ann Arbor, MI, USA) and compared with the sequences of *NF2* (NM_016418) and *NF1* (NM_001042492.3) in **GenBank**. Mutations were described according to the standard nomenclature for DNA sequence changes reported by the Human Genome Variation Society (HGVS).

### 2.3 TMT-based quantitative proteomic analysis

The tumor tissues were lysed, digested, and the resulting peptides were labeled with TMT according to the manufacturer’s protocol, with slight modifications. The TMT-labeled peptides were subsequently separated and analyzed using a nano-UPLC (EASY-nLC 1200) coupled to a Q-Exactive mass spectrometer (Thermo Finnigan). Separation was performed on a reversed-phase column (Acclaim PepMap RSLC, Thermo Scientific). Mobile phase A consisted of 0.1% formic acid and 2% acetonitrile, while mobile phase B consisted of 80% acetonitrile and 0.1% formic acid. A full MS scan was acquired with an Orbitrap at a resolution of 70,000, and MS/MS scans were performed in data-dependent mode using 32% normalized collision energy.

The MS/MS data were processed using MaxQuant software with an integrated Andromeda search engine (v.1.5.6.0). The protein sequence database (Uniprot_organism_2016_09) was downloaded from UniProt. Both the database and its reverse decoy database were searched using MaxQuant software. Trypsin was set as the specific enzyme with up to three missed cleavages, oxidation [M] and acetylation [protein N-term] were considered variable modifications (with a maximum of three modifications per peptide), and carbamidomethyl [C] was set as a fixed modification. False discovery rate (FDR) thresholds for proteins, peptides, and modification sites were set at 1%. Only unique peptides without any posttranslational modifications were included for quantification to avoid interference from variable modifications. For quantification analysis, proteins were quantified on the basis of normalized summed peptide intensities. Proteins were considered significantly differentially expressed if the TMT ratios (tumor samples vs. nerve control samples) were > 1.5 or < 0.67, with a P value < 0.05. Statistical significance was assessed using a paired t test (P < 0.05).

### 2.4 Protein–protein interaction and functional enrichment analyses

The Search Tool for the Retrieval of Interacting Genes/Proteins (STRING) database is a curated knowledge database and is used to illustrate the predicted interactions of identified proteins and interacting proteins. The proteins that were significantly differentially expressed in tumors relative to controls were processed using STRING v11.0 (https://string-db.org/) to obtain high-confidence interaction data (scores ≥ 0.7). The PPI network was visualized using the Cytoscape 3.2.1 software (https://cytoscape.org/). The Kyoto Encyclopedia of Genes and Genomes (KEGG) database was used to annotate the protein pathways. Gene Ontology (GO) analysis, including biological processes (BPs), cellular components (CCs), and molecular functions (MFs), was used to identify biological functions, processes, or cellular components for proteomics data. For functional annotation of proteins, KEGG pathway enrichment analysis and GO term annotation were performed using the DAVID bioinformatics database (https://xiantaozi.com). To address the issue of multiple testing bias, false discovery rate (FDR) correction was applied to adjust all P values, with a corrected FDR threshold of < 0.05 considered to indicate statistical significance. The background gene set used for enrichment analysis was the entire set of proteins identified in the current MS/MS proteomic profiling experiment, which ensured that the enrichment results were relevant to the experimental system and avoided false-positive associations due to inappropriate background selection.

### 2.5 Western blotting

Tumor tissues or cells were ultrasonicated in RIPA lysis buffer (Beyotime Biotechnology, Cat. No. P0013B; Shanghai, China) supplemented with 1 mM phenylmethanesulfonyl fluoride (PMSF). Sonication was performed under the following conditions: 40% amplitude, 30-second on cycles, and 30-second off cycles, with a total duration of 5 minutes on ice to prevent protein degradation. The tissue or cell extracts were subjected to Western blotting using anti-neurofibromin (#ab128054; Abcam), anti-Merlin (#HPA003097; Sigma–Aldrich), anti-EPB41L3 (#SAB1404955; Sigma–Aldrich), anti-Cyclin D1 (#55506; Cell Signaling Technology), and anti-β-actin (#A1978; Sigma–Aldrich) antibodies. The blots were visualized using Immobilon Western Chemiluminescent HRP Substrate (#WBKLS0500, Millipore). The band intensities were quantified using Image Lab software (Bio-Rad) and normalized to that of the loading control, β-actin.

### 2.6 Coimmunoprecipitation

Coimmunoprecipitation (Co-IP) was performed as follows to ensure reproducibility. Briefly, total cell lysates were obtained from human Schwann cells (HSCs) (this protocol applies to all Co-IP experiments in the manuscript) using mild RIPA lysis buffer (Cell Signaling Technology, Danvers, MA, USA; Cat. No. 9803S), a standardized nondenaturing lysis buffer suitable for preserving protein–protein interactions. This lysate was then incubated overnight at 4 °C with 2 μg of primary antibody, which had been prebound to 50% protein A/G magnetic beads twice. The specific antibodies used for Co-IP were as follows: anti-neurofibromin (rabbit, Abcam, Cat. No. ab128054), anti-Merlin (rabbit, Sigma–Aldrich, Cat. No. HPA003097), and anti-EPB41L3 (rabbit, Sigma–Aldrich, Cat. No. SAB1404955). Following this incubation, the magnetic beads were collected and subjected to four washing steps with the same mild RIPA lysis buffer (Cell Signaling Technology, #9803S), with each wash lasting 5–10 minutes at 4 °C to remove nonspecifically bound proteins. The proteins bound to the beads were subsequently eluted using 1× SDS loading buffer (containing 2% SDS, 10% glycerol, 50 mM Tris-HCl [pH 6.8], and 5% β-mercaptoethanol as a reducing agent). The eluted samples were boiled at 95 °C for 5 minutes to denature the proteins and were then analyzed by immunoblotting (Western blotting) as described previously. To ensure the specificity and reliability of the Co-IP results, the following control groups were included in all experiments: an IgG isotype control (corresponding to the species of primary antibodies, to rule out nonspecific binding), an input lysate control (10% of the total lysate used for Co-IP, to confirm the presence of target proteins in the lysate), and a bead-only control (incubation of beads with lysis buffer without primary antibody, to exclude nonspecific binding of proteins to the beads).

### 2.7 Cell culture and immunofluorescence

Human Schwann cells (HSCs) were purchased from ScienCell Research Laboratories (Cat. No. 1700; Carlsbad, CA, USA) and cultured in Schwann cell medium (ScienCell, Cat. No. 1701). The HEI-193 cell line was a generous gift from Hongsai Chen. To establish *in vitro* cultures of NF1-associated neurofibroma tissues (NF1 refers to the neurofibromatosis type 1 gene, and the tissue used herein is NF1-associated neurofibroma tissue, from which primary Schwann cells were isolated), the tumor tissues were cut into 1 mm³ segments, washed with phosphate-buffered saline (PBS), and then incubated in 0.25% trypsin for 3 hours at 37 °C with constant shaking. This extended trypsinization time (3 hours) was optimized in our laboratory to achieve sufficient dissociation of NF1-associated neurofibroma tissue, which is relatively dense compared with normal tissues (modified from the protocol described in for tissue dissociation of neurofibromas). After enzymatic incubation, the cell suspension was centrifuged at 1000 × g for 5 minutes at 4 °C, after which the cell pellet was collected. The cell pellet was then resuspended in Schwann cell medium (ScienCell, Cat. No. 1701). The cell suspension was passed through a 200-mesh filter screen (pore size: 75 μm; manufacturer: Shanghai XinYa Purification Equipment Co., Ltd., China) to remove undissociated tissue debris, and the cells were ultimately transferred to 6-cm culture dishes for subsequent culture.

For immunofluorescence (IF) staining, cells grown on glass slides were fixed with 4% paraformaldehyde (PFA) for 15 minutes at room temperature, permeabilized with 0.3% Triton X-100 (in PBS) for 10 minutes, and then blocked with 5% bovine serum albumin (BSA, dissolved in PBS) for 1 hour at room temperature to reduce nonspecific binding. The cells were subsequently incubated with primary antibodies at the following dilutions: anti-neurofibromin (#ab128054, Abcam, 1:500 dilution), anti-Merlin (#HPA003097, Sigma–Aldrich, 1:400 dilution), and anti-EPB41L3 (#SAB1404955, Sigma–Aldrich, 1:400 dilution), all of which were incubated overnight at 4 °C. After three washes with PBS (5 minutes per wash), the cells were incubated with secondary antibodies for 1 hour at room temperature in the dark. The secondary antibodies used were Alexa Fluor 488-conjugated goat anti-rabbit IgG (Thermo Fisher Scientific, Cat. No. A-11008; species specificity: goat anti-rabbit; 1:1000 dilution) and Alexa Fluor 555-conjugated goat anti-mouse IgG (Thermo Fisher Scientific, Cat. No. A-21422; species specificity: goat anti-mouse; 1:1000 dilution). The nuclei were counterstained with 4’,6-diamidino-2-phenylindole (DAPI; #1985274; Thermo Fisher Scientific) at a 1:1000 dilution for 5 minutes at room temperature. Images were acquired using a confocal microscope (LSM 880; Carl Zeiss GmbH, Germany) with a 40× oil immersion objective (numerical aperture: 1.4). Laser wavelengths were set as follows: 405 nm for DAPI, 488 nm for Alexa Fluor 488, and 555 nm for Alexa Fluor 555. The acquisition settings were consistent across all the samples, with a pixel size of 1024 × 1024 and a scan speed of 1.59 μs/pixel.

### 2.8 EdU assay

A 5-ethyl-2’-deoxyuridine (EdU) incorporation assay was performed using an EdU detection kit (Beyotime Biotechnology Co., Ltd., Shanghai, China; Cat. No. C0071S). All experiments were performed with 3 independent biological replicates and 3 technical replicates per biological replicate, and the experiments were repeated independently three times to ensure reliability. Briefly, human Schwann cells (HSCs) were seeded at a density of 1 × 10^5^ cells/well in a 24-well plate and incubated for 24 hours. Subsequently, the EdU reagent (10 μM) was added to each well, and the cells were further incubated at 37 °C for 2 hours. After incubation, the cells were processed according to the manufacturer’s instructions provided with the EdU detection kit. Finally, images were captured using a Nikon Eclipse C1 inverted fluorescence microscope (Nikon Corporation, Tokyo, Japan) with a 20× objective lens (numerical aperture: 0.5). The imaging parameters were set as follows: for EdU-positive signals, an excitation wavelength of 550 nm and an emission wavelength of 570 nm; for DAPI nuclear staining, an excitation wavelength of 358 nm and an emission wavelength of 461 nm. The exposure time was consistently set to 200 ms for all the samples, and the acquisition resolution was 1024 × 1024 pixels. The percentage of EdU-positive cells was calculated using ImageJ software (version 1.8.0). The quantification criteria were defined as cells with distinct EdU fluorescent signals (exceeding 2-fold the background fluorescence intensity) in the cytoplasm/nucleus, with DAPI staining confirming intact cell nuclei. For each sample, 5 random fields of view were selected and counted, and the percentage of EdU-positive cells was calculated as (number of EdU-positive cells/total number of DAPI-stained cells) × 100%.

### 2.9 Cell viability

Cell viability was assessed using a Cell Counting Kit-8 (CCK-8; Dojindo Laboratories, Tokyo, Japan; Cat. No. CK04). All experiments were performed with 3 independent biological replicates and 3 technical replicates per biological replicate, and the experiments were repeated independently three times to ensure reliability. Specifically, human Schwann cells (HSCs) were seeded at a density of 5 × 10³ cells/well in a 96-well plate and cultured until they adhered. After treatment (consistent with the treatment conditions used in the EdU assay), CCK-8 reagent was added to each well, and the cells were further incubated at 37 °C for 2 hours. The optical density (OD) value was then measured at 450 nm using a Thermo Scientific Multiskan FC Microplate Photometer (Thermo Fisher Scientific, Waltham, MA, USA).

### 2.10 Loss of heterozygosity analyses

Loss of heterozygosity (LOH) in the EPB41L3 gene-associated region was analyzed using the following 8 microsatellite markers: EPB41L3-SSR1, EPB41L3-SSR2, EPB41L3-SSR3, EPB41L3-SSR4, EPB41L3-SSR5, EPB41L3-SSR6, EPB41L3-SSR7, and D18S481 (sequence data are shown in **Supplementary Data 1**). PCR was performed in a 15 μL mixture containing 1.5 μL of GeneAmp Buffer II, 1.5 μL of 2 mM dNTPs, 1.5–2.5 mM MgCl_2_, 0.12 μL of 5 U/μL AmpliTaq Gold (all from Applied Biosystems, Foster City, CA, USA), 7.38–7.98 μL of H_2_O, 1.0 μL of each primer, and 1.0 μL of sample. The mixture was heated at 95 °C for 6 minutes and then cycled 40 times in a Program Temp Control System (ASTEC, Tokyo, Japan). Each cycle consisted of 30 seconds at 94 °C for denaturation, 30 seconds at 57–60 °C for annealing, and 60 seconds at 72 °C for elongation, followed by a final elongation step at 72 °C for 10 minutes. The samples were electrophoresed using an Applied Biosystems 3130/3130xl Genetic Analyzer or Applied Biosystems 377 DNA Sequencer. LOH analysis was performed using GeneMapper 4.0 or GeneScan 3.1 (both from Applied Biosystems). To confirm reproducibility, all the samples were examined at least twice. A reduction in signal intensity exceeding 50% was defined as LOH.

### 2.11 Cell culture and generation of EPB41L3 knockout cells

Short hairpin RNA (shRNA) targeting EPB41L3 and a negative control shRNA were designed and synthesized by GenePharma Co., Ltd. (Shanghai, China). The shRNA sequences were subcloned and inserted into the pLKO.1 vector (Takara Bio, Kusatsu, Japan). For lentivirus production, the recombinant vectors were transfected into HEK293T cells. Schwann cells were then infected with either control or EPB41L3-shRNA lentivirus, followed by selection with 1.0 μg/mL puromycin (Sigma) for 2 weeks. Stable cell lines were maintained in Schwann cell medium. The interference efficiency of EPB41L3 was verified by quantitative real-time polymerase chain reaction (qRT−PCR) and Western blot analysis.

The sequences of shRNA-EPB41L3 were as follows:
sense, 5’-GCUGCGAAUAAACAGAUUUTT-3’;
antisense, 5’-AAAUCUGUUUAUUC GCAGCTT-3’.
The sequences of the negative control shRNA were as follows:
sense, 5’-CUGCGGAAAUAAUUUCAGATT-3’;
antisense, 5’-AAUCAUUUUGACGUUAGCCTT-3’.

### 2.12 Statistical analysis

Statistical analysis was performed using IBM SPSS Statistics software (Version 26.0; IBM Corp., Armonk, NY, USA). The data are presented as the mean ± standard deviation (SD) of at least three independent biological replicates, and each biological replicate contained three technical replicates. Independent biological replicates were defined as experiments performed on different days using distinct cell passages and freshly prepared reagents. Differences between two groups were analyzed using Student’s t test, while comparisons among multiple groups were evaluated by one-way analysis of variance (ANOVA) followed by Tukey’s *post hoc* test. No further adjustments for multiple comparisons were made beyond *post hoc* testing, as the comparisons were restricted to *a priori* defined experimental groups with clear biological relevance. A value of P < 0.05 was considered to indicate statistical significance.

## 3 Results

### 3.1 Mutation analyses of neurofibromatosis types 1 and 2

Mutational analysis of tumor samples associated with *NF1* revealed that all samples harbored at least one variant within the *NF1* gene. Among the five NF1 tumor samples analyzed, two samples (T101 and T105) contained synonymous variants, whereas the remaining three samples carried pathogenic variants (**Table 1**). Evolutionary conservation analysis was performed using ConSurf. Amino acid conservation scores were calculated and classified into nine grades, as indicated by the color scale shown in **Supplementary Figure 1**. The positions of the identified variants were mapped onto the protein structure using PyMOL (21). **Supplementary Figure 1A** illustrates the locations of the variants identified in NF1, while **Supplementary Figure 1B** shows the locations of the variants identified in NF2. All variant positions are indicated by red spheres, with the corresponding amino acid positions labeled. Sequence analysis of NF2 tumor samples revealed that 8 of 10 patients carried clinically significant variants in the *NF2* gene, whereas two samples did not present detectable variants (**Table 1**). On the basis of these results, samples carrying pathogenic variants were selected for subsequent analyses.

**Table 1:** Genetic information for neurofibromatosis type 1 and type 2.

Case	Diagnosis	*NF2* gene point mutation				*NF1* gene point mutation				Loss of heterozygosity analysis for *EPB41L3*
		Exon	Sequence alteration	Codon	Consequence	Exon	Sequence alteration	Codon	Consequence	
T101	NF1	/	/	/	/	E18	c.2034G>A	p.P678P	synonymous	Lost(D18S481)
T102	NF1	/	/	/	/	E18	c.2034G>A	p.P678P	synonymous	Retention
						E07	c.702G>A	p.L234L	synonymous
						E17	c.1986 del A		framshift
T103	NF1	/	/	/	/	E18	c.2034G>A	p.P678P	synonymous	Lost(EPB41L3-SSR2)
						E07	c.702G>A	p.L234L	synonymous
		E30					c.4084C>T	p.R1362-	nonsense
T104	NF1	/	/	/	/	E18	c.2034G>A	p.P678P	synonymous	Retention
						E25	c.3311T>G	p.L1104R	missense
T105	NF1	/	/	/	/	E07	c.702G>A	p.L234L	synonymous	Lost(EPB41L3-SSR2)
		Lost(EPB41L3-SSR7)							
T468	NF2	E12	c.1159A>G	p.K387E	missense	/	/	/	/	Retention
T469	NF2	E06	c.528_529del GT		framshift	/	/	/	/	Lost(EPB41L3-SSR3)
T470	NF2	E06	c.592C>T	p.R198-	nonsense	/	/	/	/	Retention
T473	NF2	None	None	None	None	/	/	/	/	Retention
T475	NF2	E02	c.235A>T	p.K79-	nonsense	/	/	/	/	Retention
T478	NF2	E13	c.1439C>T	p.T480M	missense	/	/	/	/	Lost(D18S481)
T480	NF2	None	None	None	None	/	/	/	/	Retention
T481	NF2	E09	c.832A>T	p.K278-	nonsense	/	/	/	/	Retention
T483	NF2	E08	c.770del C		framshift	/	/	/	/	Retention
T488	NF2	E07	c.646del A		framshift	/	/	/	/	Retention

### 3.2 Proteomic analysis of neurofibromatosis type 1

**Figure 1:**
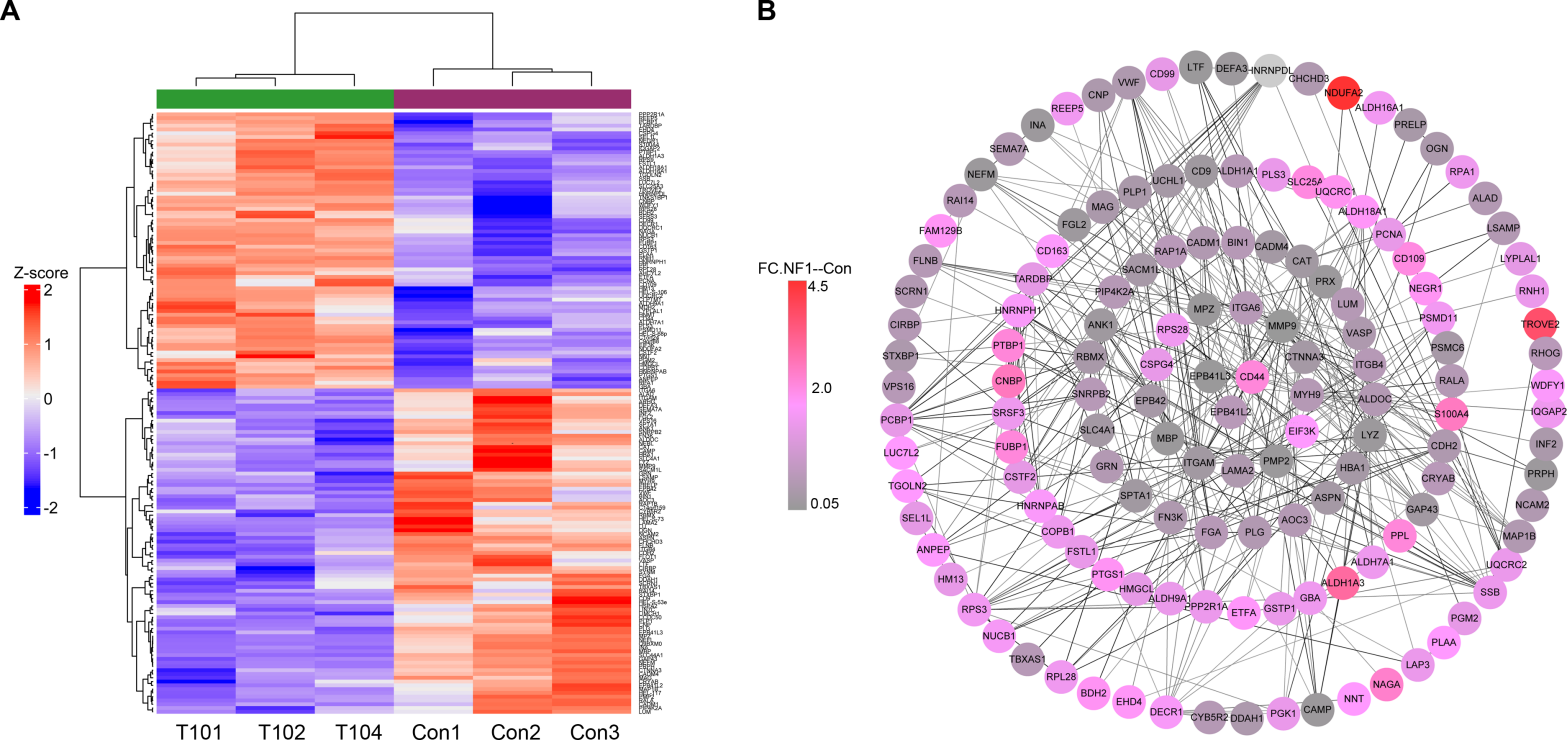
Proteomic analysis of differentially expressed proteins between neurofibromatosis type 1 patients and nerve tissue controls (CON). **(a)** Hierarchically clustered heatmaps of the 159 significantly deregulated proteins in 3 samples from each group. Red represents a high z score, and blue represents a low z score; the “row names” NF1.1–3 indicate the tumors T101, T102 and T104. **(b)** Protein–protein interaction (PPI) network of the above deregulated proteins. Circular nodes denote proteins, and their connecting edges denote protein–protein interactions. The node size is proportional to the fold change of the corresponding modulated protein.

Using label-free LC–MS proteomic analysis, compared with normal nerve tissue samples, a total of 159 proteins, including 73 upregulated and 86 downregulated proteins (**Supplementary Table 2**; **Figure 1A**), were identified as being differentially expressed in *NF1* tumor samples. The top 10 upregulated and downregulated proteins are listed in **Table 2**. Compared with normal control samples, several members of the aldehyde dehydrogenase (ALDH) superfamily, including ALDH1A3, ALDH18A1, ALDH16A1, ALDH7A1, and ALDH9A1, were upregulated in NF1 tumor samples (**Supplementary Table 2**). In contrast, matrix metallopeptidase 9 (MMP9) was among the downregulated proteins identified in the NF1 samples. In addition, increased expression of CD44 was observed in both NF1 and NF2 tumor samples. EPB41L3 was also downregulated across most neurofibromatosis sample types. Protein–protein interaction (PPI) analysis of the differentially expressed proteins is shown in **Figure 1B**, revealing a highly connected interaction cluster centered on CD44 and associated proteins, including PML, CD9, CD34, LAMA2, LAMB1, ALDH1A1, ITGAV, GBA, RDX, DCN, and NID1.

**Table 2:** The top 10 proteins up-regulated or down-regulated in NF1 compared with nerve control.

Gene.names	pv.NF1--CON	fc.NF1--CON	Annotation
MMP9	0.045	0.045	Matrix metallopeptidase 9
EPB41L3	0.005	0.074	Erythrocyte membrane protein band 4.1-like 3
MBP	0.004	0.076	Myelin basic protein
PMP2	0.025	0.085	Peripheral myelin protein 2
MPZ	0.000	0.088	Myelin protein zero
LTF	0.026	0.090	Lactotransferrin
LYZ	0.038	0.099	Lysozyme
PRX	0.002	0.100	Periaxin
NEFM	0.002	0.104	Neurofilament, medium polypeptide
CAMP	0.038	0.105	Cathelicidin antimicrobial peptide
CD44	0.007	2.422	CD44 molecule
PTBP1	0.004	2.433	Polypyrimidine tract binding protein 1
HNMT	0.038	2.499	Histamine N-methyltransferase
NAGA	0.019	2.581	N-acetylgalactosaminidase, alpha
S100A4	0.005	2.712	S100 calcium binding protein A4
CNBP	0.021	2.748	CCHC-type zinc finger, nucleic acid binding protein
C1orf24	0.004	2.858	Family with sequence similarity 129, member A
ALDH1A3	0.006	3.087	Aldehyde dehydrogenase 1 family, member A3
TROVE2	0.005	3.695	TROVE domain family, member 2
NDUFA2	0.001	4.331	NADH dehydrogenase (ubiquinone) 1 alpha subcomplex 2

**Figure 2:**
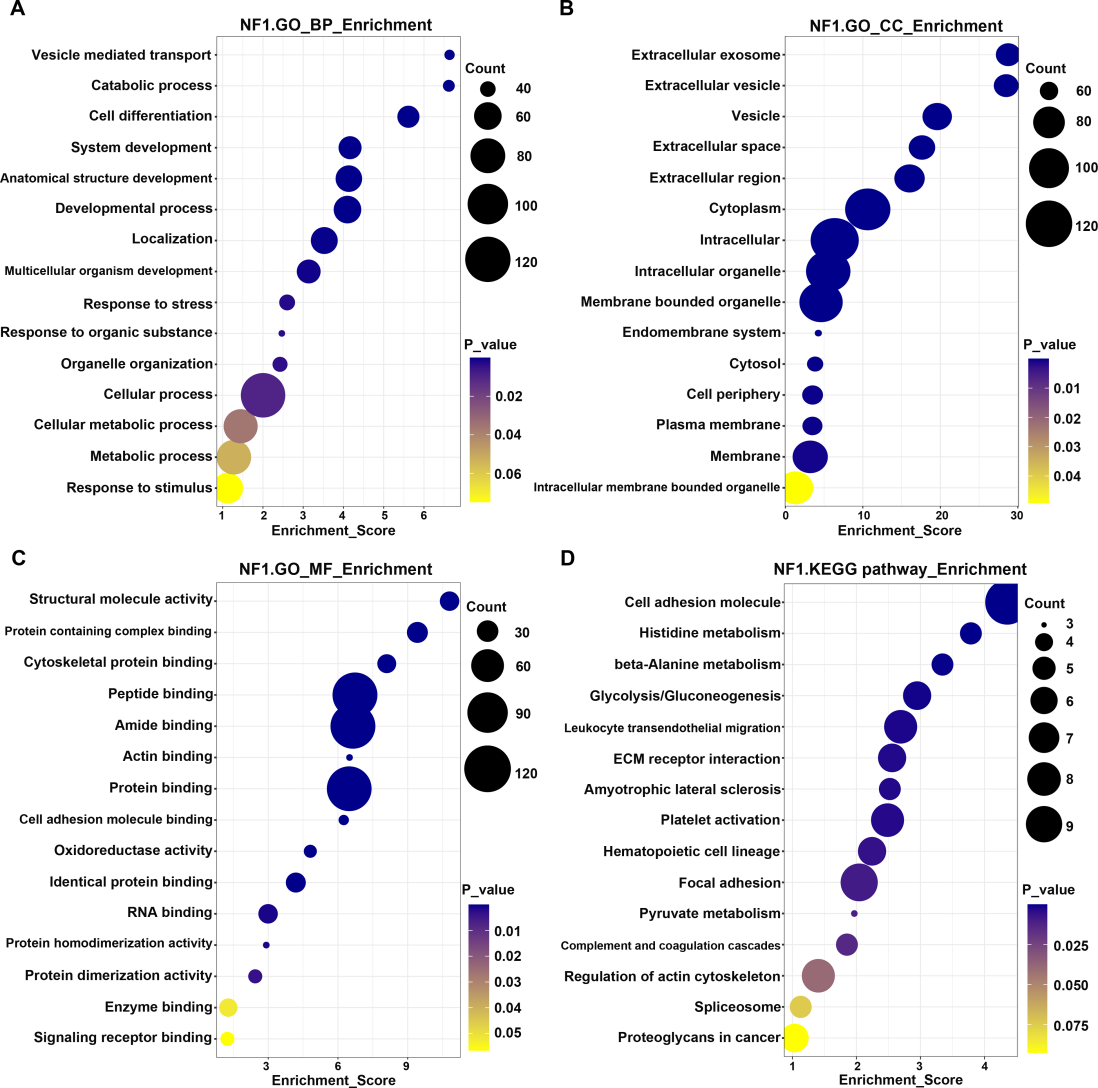
Enrichment analysis of GO terms and KEGG pathways for neurofibromatosis type 1. **(a-c)** The GO terms in the biological process **(a)**, cellular component **(b)** and molecular function **(c)** categories are depicted. **(d)** Bubble chart depicting the most frequently enriched pathways corresponding to the deregulated proteins. The color of each dot denotes the *P* value, and the size of each dot denotes the number of proteins involved in each pathway.

Functional enrichment analysis of the differentially expressed proteins was performed using Gene Ontology and KEGG pathway analyses (**Figures 2A–D**). Enriched categories included biological processes, cellular components, and molecular functions related to cell adhesion and histidine metabolism.

### 3.3 Proteomic analysis of neurofibromatosis type 2

**Figure 3:**
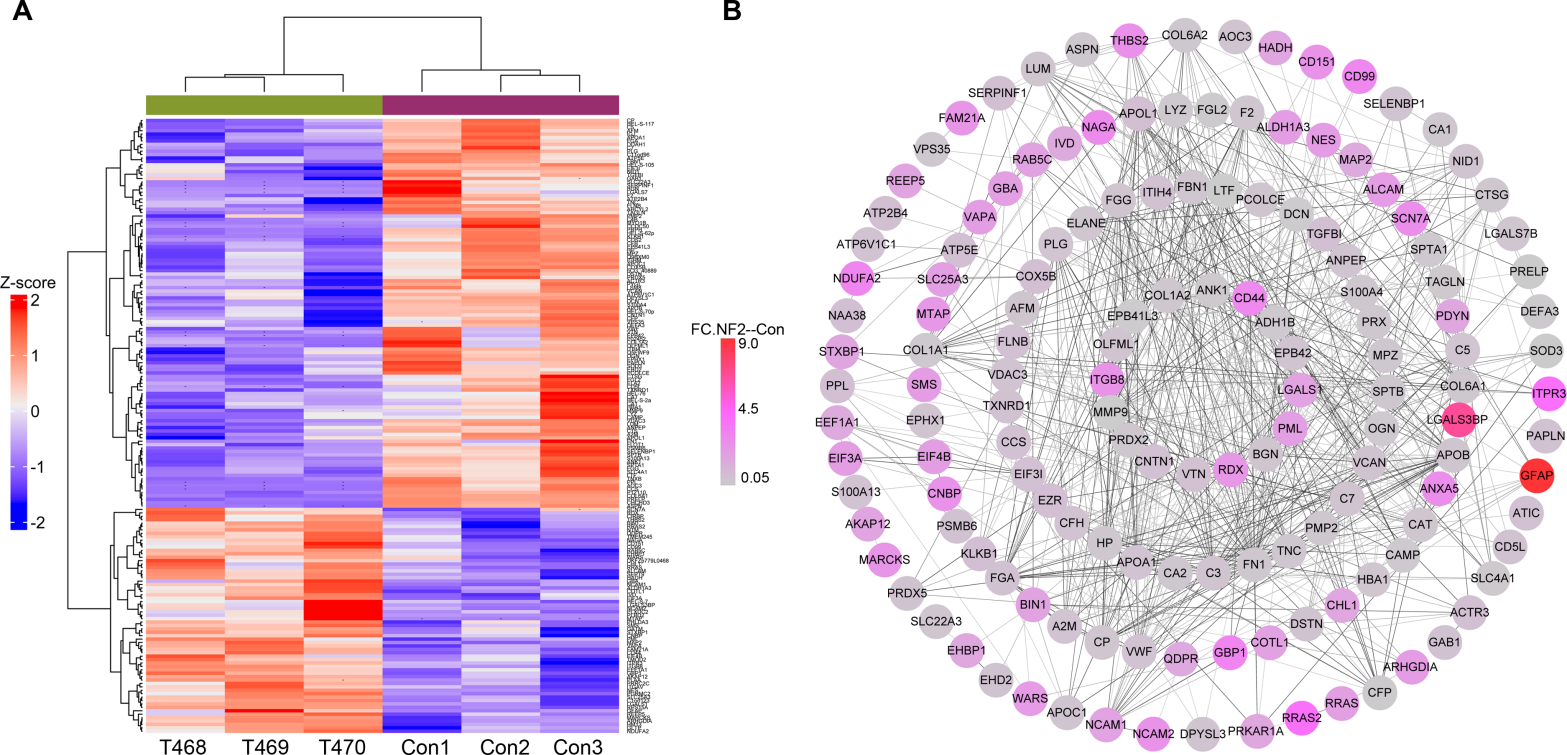
Proteomic analysis of differentially expressed proteins between neurofibromatosis type 2 patients and nerve tissue controls (CON). **(a)** Hierarchically clustered heatmaps of the 180 significantly deregulated proteins in 3 samples from each group. Red represents a high z score, and blue represents a low z score; “row names” NF2.1–3 indicate tumors T468, T469 and T470. **(b)** Protein–protein interaction (PPI) network of the above deregulated proteins. Circular nodes denote proteins, and their connecting edges denote protein–protein interactions. The node size is proportional to the fold change of the corresponding modulated protein.

Compared with normal nerve tissues, a total of 180 proteins were identified as significantly differentially expressed in NF2 tumor samples, including 66 upregulated and 114 downregulated proteins (**Supplementary Table 3**; **Figure 3A**). The top 10 upregulated and downregulated proteins are listed in **Table 3**. Among the upregulated proteins, glial fibrillary acidic protein (GFAP) had the highest relative expression level in NF2 tumor samples. In addition, decreased expression of EPB41L3 was observed in NF2 tumors, which is consistent with the findings obtained in NF1 samples. Increased expression of CD44 was also detected in NF2 tumor tissues.

**Table 3:** The top 10 proteins up-regulated or down-regulated in NF2 compared with nerve control.

Gene names	pv..NF2--CON	fc..NF2–CON	Annotation
MMP9	0.012	0.053	Matrix metallopeptidase 9
LTF	0.001	0.058	Lactotransferrin
PRELP	0.000	0.088	Proline/arginine-rich end leucine-rich repeat protein
SOD3	0.002	0.089	Superoxide dismutase 3
PRPH	0.000	0.092	Peripherin
EPB41L3	0.001	0.096	Erythrocyte membrane protein band 4.1-like 3
DCN	0.000	0.105	Decorin
DEFA3	0.007	0.123	Defensin,alpha 3,neutrophil-specific
CFP	0.008	0.125	Complement factor properdin
FGL2	0.004	0.130	Fibrinogen-like 2
NAGA	0.013	2.907	N-acetylgalactosaminidase, alpha
CD44	0.003	3.000	CD44 molecule
CD99	0.010	3.015	CD99 molecule
NDUFA2	0.007	3.132	NADH dehydrogenase (ubiquinone) 1 alpha subcomplex,2
GBP1	0.004	3.218	Guanylate binding protein 1
RRAS2	0.009	4.020	Related RAS viral (r-ras) oncogene homolog 2
ITPR3	0.005	4.072	Inositol 1,4,5-trisphosphate receptor, type 3
PLBD2	0.034	5.642	Phospholipase B domain containing 2
LGALS3BP	0.018	6.774	Lectin, galactoside-binding, soluble, 3 binding protein
GFAP	0.014	8.904	Glial fibrillary acidic protein

**Figure 4:**
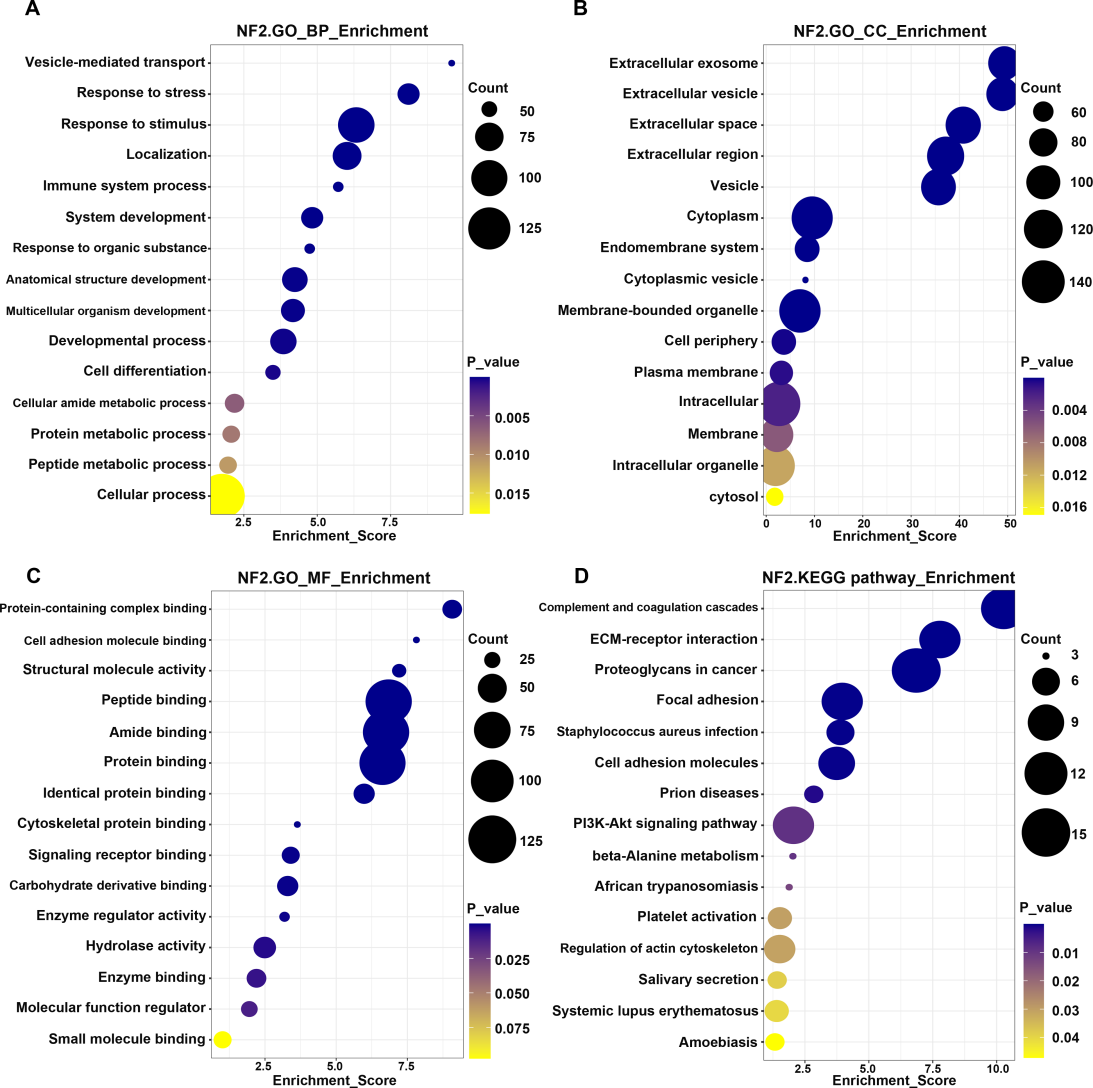
Enrichment analysis of GO terms and KEGG pathways for neurofibromatosis type 2. **(a–c)** The GO terms in the biological process **(a)**, cellular component **(b)** and molecular function **(c)** categories are depicted. **(d)** Bubble chart depicts the most frequently enriched pathways corresponding to the deregulated proteins. The color of each dot denotes the *P* value, and the size of each dot denotes the number of proteins involved in each pathway.

Protein–protein interaction (PPI) analysis of the differentially expressed proteins is shown in **Figure 3B**. Functional enrichment analysis of the differentially expressed proteins was performed using Gene Ontology and KEGG pathway analyses (**Figures 4A–D**). Enriched categories included biological processes, cellular components, and molecular functions related to complement and coagulation cascades, regulation of the actin cytoskeleton, and extracellular matrix–receptor interactions.

### 3.4 Common neurofibromatosis type 1 and neurofibromatosis type 2 proteins

**Figure 5:**
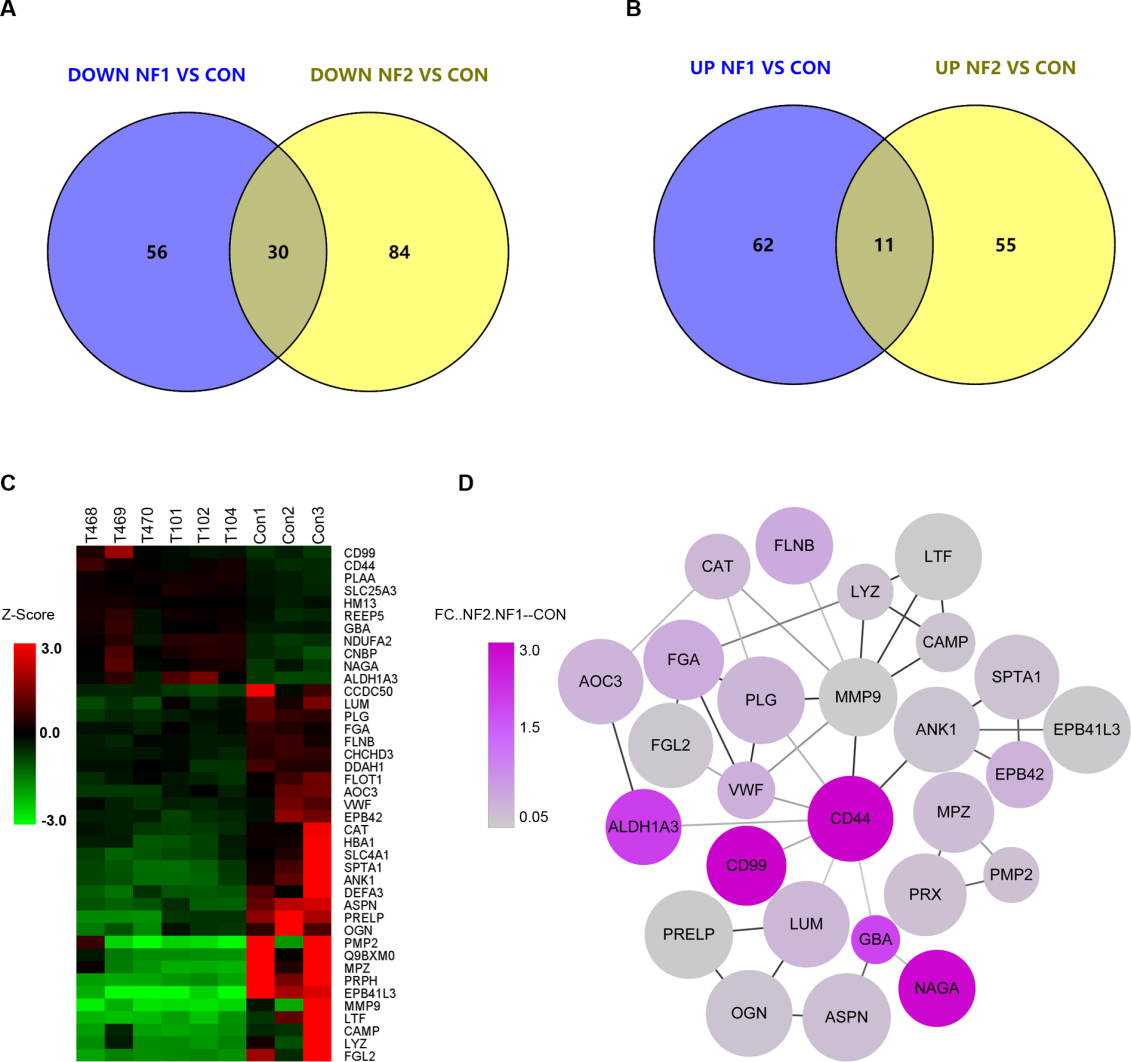
Common differentially expressed proteins in neurofibromatosis type 1 and neurofibromatosis type 2. **(a)** Thirty common downregulated proteins identified by Venn software. **(b)** Eleven common upregulated proteins identified by Venn software. **(c)** Hierarchically clustered heatmaps of the 41 significantly common deregulated proteins in 6 samples from each group. Red represents a high z score, and blue represents a low z score; the “row names” NF1.1–3 indicate tumors T101, T102 and T104, respectively; and NF2.1–3 indicate tumors T468, T469 and T470, respectively. **(d)** Protein–protein interaction (PPI) network of the above deregulated proteins. Circular nodes denote proteins, and their connecting edges denote protein–protein interactions. The node size is proportional to the fold change of the corresponding modulated protein.

Comparative analysis of proteins with significantly altered expression in NF1 and NF2 tumor samples relative to that in normal nerve tissue controls revealed 11 proteins that were commonly upregulated and 30 proteins that were commonly downregulated in both tumor types (**Figures 5A, B**). Among the commonly downregulated proteins, EPB41L3 was consistently detected in both NF1 and NF2 tumor samples (**Figure 5C**). In addition, protein–protein interaction analysis revealed CD44 as a highly connected node within the shared interaction network between the NF1 and NF2 samples (**Figure 5D**).

### 3.5 Expression of EPB41L3, merlin, and neurofibromin in primary cultures and tumor tissue

**Figure 6:**
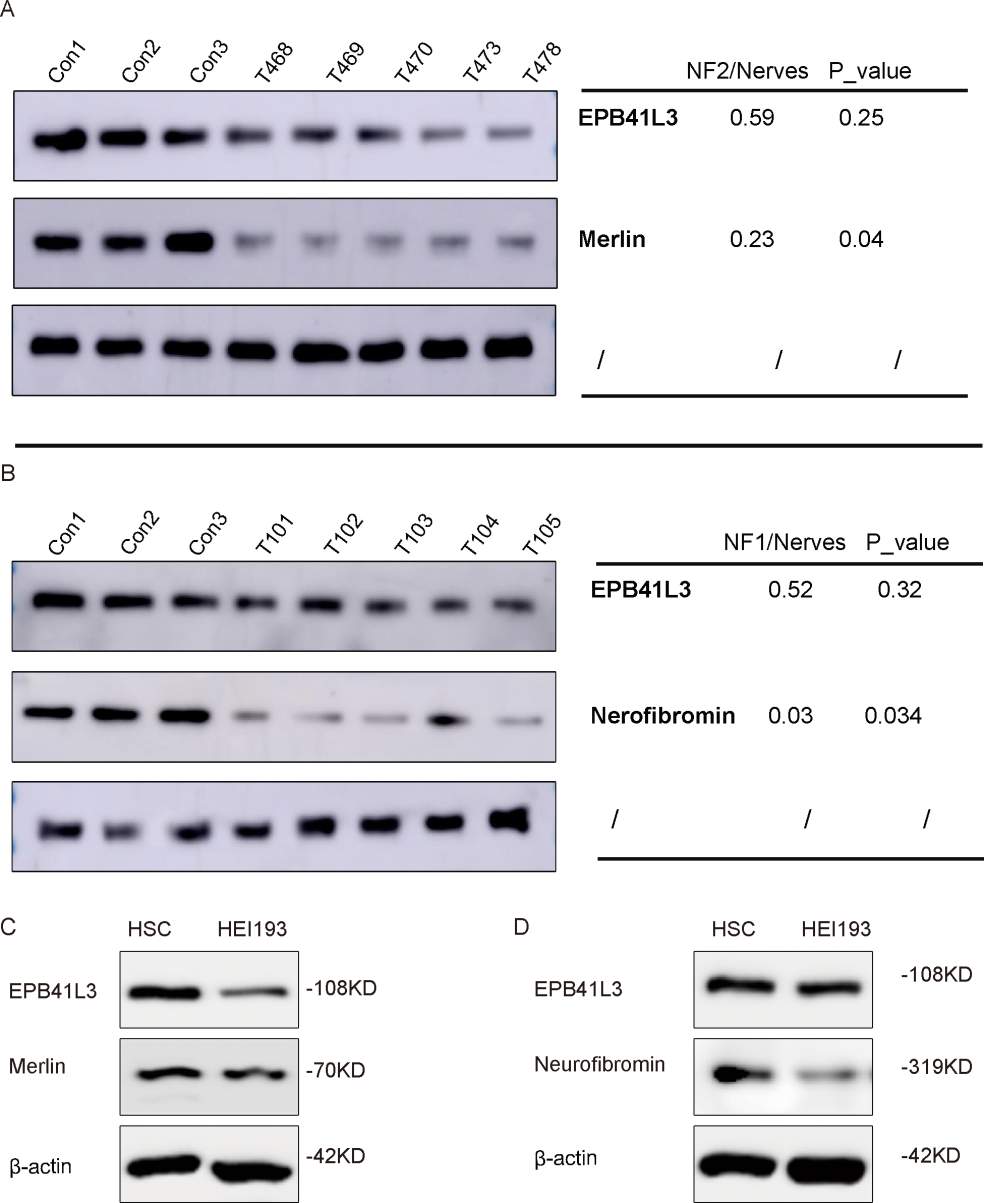
The expression of EPB41L3, Merlin and neurofibromin in NF1 and NF2 patients. **(A, B)** Validation of the differential expression of EPB41L3, Merlin and neurofibromin in NF1 and NF2 patients compared with those in nerve tissue controls by Western blotting. The band intensities of the target proteins in NF1 and NF2 are expressed relative to those in the nerve controls to obtain relative grayscale values. **(C, D)** Western blot analysis of EPB41L3, Merlin, neurofibromin protein expression in HEI193 cells compared with that in HSCs.

Western blotting was performed to assess the expression levels of EPB41L3 and selected proteins of interest, including Merlin (encoded by *NF2*) and neurofibromin (encoded by *NF1*), in tumor tissues and primary cell cultures (**Figures 6A–D**). Quantitative analysis revealed a significant reduction in EPB41L3 protein levels in NF2 tumor samples compared with those in normal nerve tissue controls, with an average decrease to approximately 0.59 relative to control levels. A similar reduction in EPB41L3 expression was observed in NF1 tumor samples, with levels reduced to approximately 0.52 relative to those in controls (**Figures 6A, B**).

In NF2 tumor samples, *Merlin* protein levels were also significantly reduced (approximately 0.23 relative to those in controls; p = 0.04), whereas neurofibromin expression was significantly decreased in NF1 tumor samples (approximately 0.03 relative to that in controls; p = 0.034) (**Figures 6A, B**).

Reduced *EPB41L3* expression was further confirmed in HEI193 cells and in primary *NF1* tumor-derived cultures. In these cellular models, decreased levels of *EPB41L3* were accompanied by reduced expression of Merlin and neurofibromin (**Figures 6C, D**).

### 3.6 Loss of heterozygosity analysis for EPB41L3

Loss of heterozygosity (LOH) analysis of the *EPB41L3* genomic region was performed using paired tumor and blood DNA samples from 15 patients, including 5 NF1 and 10 NF2 patients. Eight highly polymorphic microsatellite markers spanning the *EPB41L3* locus (*EPB41L3*-SSR1 to *EPB41L3*-SSR7 and D18S481) were analyzed (**Supplementary Data 1**).

LOH was detected at four microsatellite loci (*EPB41L3*-SSR2, *EPB41L3*-SSR3, *EPB41L3*-SSR7, and D18S481) (**Supplementary Figure 2**). Among the 15 tumors analyzed, 10 samples retained both alleles, whereas 5 samples exhibited LOH compared with the corresponding blood DNA, corresponding to an overall LOH frequency of 33.3%. When stratified by tumor type, LOH was observed in 3 of 5 NF1 tumors (60%) and in 2 of 10 NF2 tumors (20%) (**Table 1**).

### 3.7 EPB41L3 inhibited proliferation in HSCs

**Figure 7:**
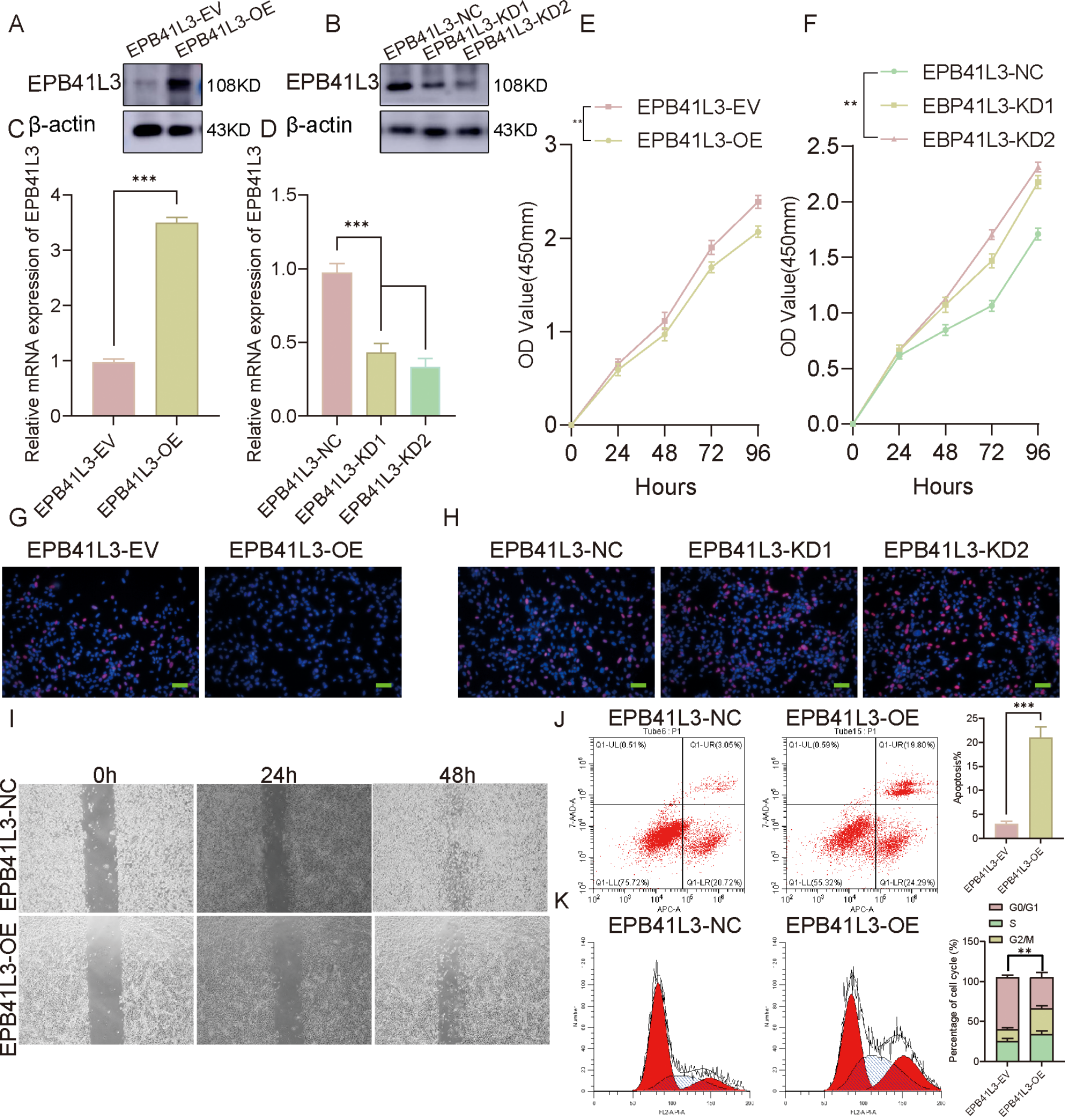
*EPB41L3* inhibited the proliferation of HEI193 cells. **(a, b)** Western blotting was used to assess the expression level of EPB41L3 (n = 3). **(c, d)** qPCR was used to investigate the expression level of *EPB41L3* mRNA in HEI193 cells (n = 3). **(e, f)** CCK8 assays revealed that *EPB41L3* decreases the viability of HEI193 cells, whereas knockdown of *EPB41L3* leads to an increase in the viability of HEI193 cells (n = 3). **(g, h)**. Representative images of EdU staining analysis are shown. *EPB41L3* overexpression significantly inhibited the proliferation of HEI193 cells. Knockdown of *EPB41L3* rescued the inhibition of HEI193 cell proliferation. Scale bar = 50 μm (n = 8). **(i)** The inhibitory effect on the migration of HEI193 cells was examined by a wound healing assay after 48 hours. Scale bar: 200 μm (n=8). **(j)** The total proportion of early apoptotic cells (annexin V+/PI−) and late apoptotic cells (annexin V+/PI+) was used to evaluate HEI193 cell apoptosis after *EPB41L3 was overexpressed* (n=3). **(k)** Representative results of the cell cycle and quantitative analyses of HEI193 cells after *EPB41L3* overexpression (n=3). The data are presented as the mean ± SD. P < 0.05; *P < 0.01 compared with controls.

To assess the effects of *EPB41L3* on cellular phenotypes, *EPB41L3* expression was modulated in HSCs through lentiviral overexpression and shRNA-mediated knockdown approaches. Changes in *EPB41L3* expression were confirmed by Western blotting and qPCR (**Figures 7A–D**). CCK-8 assays revealed reduced cell viability following *EPB41L3* overexpression, whereas *EPB41L3* knockdown was associated with increased cell viability (**Figures 7E, F**). Consistently, the results of the EdU incorporation assays revealed a decreased proportion of EdU-positive *EPB41L3*-overexpressing HSCs and an increased proportion of *EPB41L3*-knockdown cells (**Figures 7G, H**). Cell migration, as assessed by scratch assays, was reduced 48 hours after *EPB41L3* overexpression (**Figure 7K**). Flow cytometric analysis revealed increased proportions of early (Annexin V^+^/PI^-^) and late (Annexin V^+^/PI^+^) apoptotic cells following *EPB41L3* overexpression (**Figure 7L**). Cell cycle analysis revealed a decrease in the proportion of cells in the G0/G1 phase and an increase in the proportion of cells in the G2/M phase following *EPB41L3* overexpression (**Figure 7M**).

**Figure 8:**
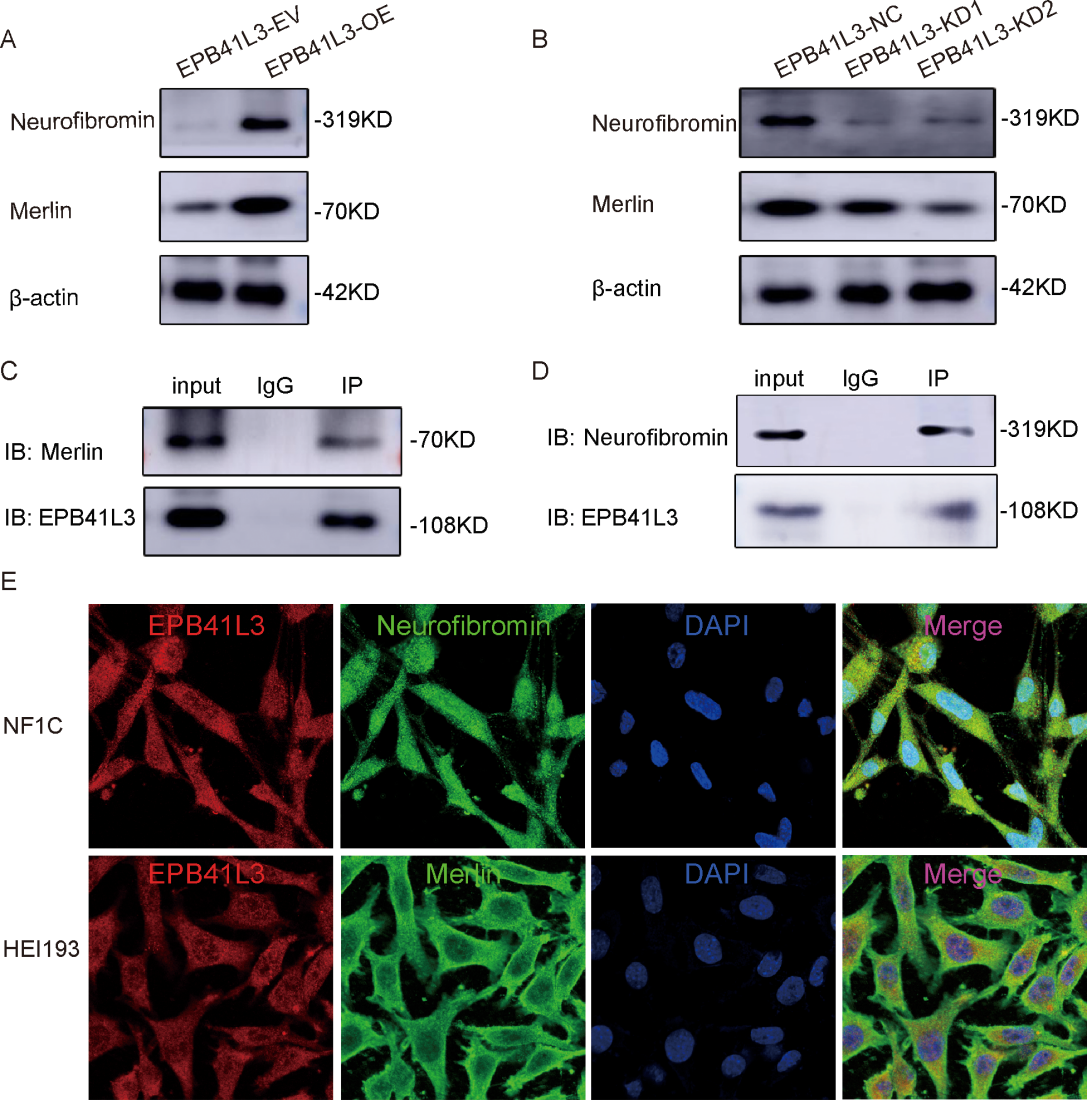
EPB41L3 interacts with Merlin and neurofibromin and upregulates their protein expression. **(A, B)** Western blotting was employed to assess the expression level of EPB41L3. **(C, D)** EPB41L3 interacts with Merlin and neurofibromin, respectively. **(E)** EPB41L3 colocalizes with both Merlin and neurofibromin in the cytoplasm.

In terms of molecular associations, *EPB41L3* overexpression was accompanied by increased levels of *Merlin* and neurofibromin, whereas *EPB41L3* knockdown was associated with reduced levels of these proteins (**Figures 8A, B**). Coimmunoprecipitation assays demonstrated interactions between endogenous EPB41L3 and Merlin, as well as between EPB41L3 and neurofibromin, in HSCs (**Figures 8C, D**). Immunofluorescence analysis revealed predominant cytoplasmic localization of EPB41L3 and Merlin, whereas neurofibromin displayed mainly nuclear localization (**Figure 8E**).

## 4 Discussion

### 4.1 Comparative proteomic alterations in NF1 and NF2 tumors

This study aimed to characterize and compare the proteomic landscapes of NF1 and NF2 tumors in relation to normal nerve tissue. Although *NF1* and *NF2* arise from distinct genetic mutations, both tumor types originate from Schwann cells and share overlapping pathological features, suggesting the existence of common dysregulated molecular pathways (1, 2). By analyzing NF1 and NF2 tumors both independently and comparatively, this study sought to identify shared proteomic alterations that may contribute to tumor biology.

High-throughput proteomic profiling revealed extensive changes in protein expression in both NF1 and NF2 tumors. As with any proteomic analysis of bulk tumor tissue, these findings should be interpreted in light of inherent limitations, including tumor heterogeneity and the presence of multiple nonneoplastic cell types (31, 32). Nevertheless, the datasets provide a valuable overview of dysregulated pathways that may be relevant to neurofibromatosis-associated tumorigenesis.

### 4.2 Cytoskeletal and membrane-associated pathways in NF1 tumors

In NF1 tumors, functional enrichment analyses revealed alterations in pathways related to cytoskeletal organization, actin binding, and cell adhesion. These findings are consistent with the established role of neurofibromin in regulating actin dynamics and cell motility through RAS-dependent and RAS-independent mechanisms (33). Dysregulation of actin cytoskeleton-related pathways has previously been associated with altered Schwann cell behavior and neurofibroma development.

Several members of the aldehyde dehydrogenase (ALDH) superfamily are upregulated in NF1 tumors. ALDH enzymes have been implicated in cellular detoxification, metabolic reprogramming, and tumor progression across multiple cancer types (22, 23). Additionally, altered expression of extracellular matrix-associated proteins, including MMP9, was observed. Although MMP9 has been linked to tumor microenvironment remodeling and early tumorigenesis in other contexts (24), its downregulation in NF1 tumors may reflect differences in tumor stage, cellular composition, or microenvironmental context.

Increased expression of CD44 was detected in NF1 tumors, in agreement with previous reports on Schwann cell-derived tumors (24, 25). CD44 is functionally linked to *Merlin* and neurofibromin signaling and is known to be influenced by RAS pathway activation (26, 27). These observations support the notion that alterations in cytoskeletal and membrane-associated protein networks are prominent features of NF1 tumor biology.

### 4.3 Cytoskeletal signaling and PI3K–Akt pathway alterations in NF2 tumors

In NF2 tumors, enrichment analyses similarly revealed the dysregulation of pathways related to cytoskeletal organization, extracellular matrix interactions, and actin filament regulation. This finding is consistent with the known function of Merlin, a FERM-domain protein closely related to the ezrin–radixin–moesin (ERM) family, which plays a critical role in linking membrane proteins to the actin cytoskeleton (28). GFAP was among the most strongly upregulated proteins in NF2 tumors. Elevated GFAP expression has been described as a characteristic feature of NF2-associated and sporadic schwannomas, reflecting alterations in glial differentiation and cytoskeletal architecture (29). In addition, dysregulation of the PI3K–Akt signaling pathway was observed, which is in line with the findings of previous studies indicating that this pathway is involved in schwannoma growth and NF2 tumorigenesis (30). Together, these findings reinforce the importance of cytoskeletal and membrane-associated signaling networks in NF2-associated tumors.

### 4.4 Shared dysregulated proteins between NF1 and NF2

Despite the distinct genetic origins of NF1 and NF2 tumors, comparative proteomic analysis revealed a subset of proteins that were consistently dysregulated in both tumor types. Among these proteins, EPB41L3 was identified as a commonly downregulated protein in NF1 and NF2 tumors. EPB41L3 is a member of the protein 4.1 family and was originally described as a cytoskeleton-associated tumor suppressor; it is also known as DAL-1 in lung cancer (31). Loss of *EPB41L3* expression has also been reported in *Merlin*-deficient meningiomas since 2005 (32), supporting the plausibility of its dysregulation in Schwann cell-derived tumors. In addition, EPB41L3 interacts with CD44 and ANK1, suggesting that EPB41L3 plays a role in membrane–cytoskeleton organization (**Figure 5D**). The identification of multiple downregulated FERM domain-containing proteins in this study further supports the relevance of this protein family in neurofibromatosis-associated tumors. These observations provide a rationale for selecting *EPB41L3* for further validation and functional analysis. .

### 4.5 Functional implications of *EPB41L3* dysregulation

Functional assays performed in HSCs demonstrated that the modulation of *EPB41L3* expression influences several cellular phenotypes *in vitro*. *EPB41L3* overexpression was associated with reduced cell viability, decreased EdU incorporation, impaired migration, increased apoptosis, and accumulation of cells in the G2/M phase. Conversely, *EPB41L3* knockdown had opposite effects, including increased proliferation and reduced apoptosis rates. Importantly, these findings indicate that *EPB41L3* is capable of modulating key cellular processes; however, they do not establish *EPB41L3* as a primary driver of tumorigenesis. The use of an *in vitro* HSC model represents a limitation, and these results should be interpreted as indicating functional capacity rather than direct evidence of *in vivo* tumor behavior.

### 4.6 Association of EPB41L3 with Merlin and neurofibromin

The observed changes in the protein levels of Merlin and neurofibromin following *EPB41L3* modulation, together with the detection of physical interactions among these proteins by coimmunoprecipitation, suggest that EPB41L3 may participate in shared protein complexes or cytoskeleton-associated networks involving known neurofibromatosis tumor suppressors.

Immunofluorescence analyses revealed predominantly cytoplasmic localization of EPB41L3 and Merlin, whereas neurofibromin was primarily localized to the nucleus, indicating spatially distinct but potentially coordinated functions. These data support an associative relationship rather than a direct regulatory hierarchy.

### 4.7 Contribution of *EPB41L3* loss of heterozygosity to NF1 and NF2

Analysis of loss of heterozygosity (LOH) at the *EPB41L3* locus revealed allelic loss in a subset of NF1- and NF2-associated tumors. Although LOH was more frequently observed in NF1 tumors in this cohort, the limited sample size precludes definitive conclusions regarding tumor type specificity. Previous studies have reported frequent LOH of *EPB41L3* in meningiomas harboring *NF2* mutations, particularly in malignant subtypes (32, 33). In this context, the present findings suggest that *EPB41L3* LOH may contribute to reduced gene expression in a subset of neurofibromatosis-associated tumors, but it should be regarded as a contributory rather than universal mechanism.

### 4.8 Limitations and perspectives

This study has several limitations, including the relatively small sample size, the use of bulk tumor tissue for proteomic analysis, and the reliance on *in vitro* cellular models for functional assays. Future studies employing larger patient cohorts, single-cell or spatial proteomic approaches, and *in vivo* models will be necessary to further clarify the functional role of *EPB41L3* and its relevance in neurofibromatosis-associated tumorigenesis.

## 5 Conclusions

In summary, our integrated proteomic and functional analyses revealed that cytoskeletal and membrane-associated pathways are commonly altered in NF1 and NF2 tumors. *EPB41L3* emerges as a shared dysregulated candidate that may participate in these networks. While the present study provides a framework for understanding common molecular alterations in neurofibromatosis-associated tumors, further work will be needed to define the precise functional role of *EPB41L3* and to evaluate its potential relevance in therapeutic contexts.

## Data Availability

The original contributions presented in the study are publicly available. This data can be found here: https://doi.org/10.6084/m9.figshare.32252877.

## Supplementary material

Supplementary Figure 1 Schematic diagram of proteins. **(A)** Consurf colored structure of the full-length human NF1 dimer. **(B)** Consurf colored structure of the full-length human NF2 dimer.

Supplementary Figure 2 Loss of heterozygosity analysis for *EPB41L3* in NF1 and NF2. Comparison of lymphocyte DNA and tumor DNA using the flanking, D18S481 and intragenic *EPB41L3*-SSR1, *EPB41L3*-SSR2, *EPB41L3*-SSR3, *EPB41L3*-SSR4, EPB41L3-SSR5, *EPB41L3*-SSR6, and *EPB41L3*-SSR7 microsatellite markers in the *EPB41L3* gene region. In this case, four markers showed loss of heterozygosity because of allele loss in the tumor DNA.

Supplementary Figure 3 *EPB41L3* knockdown downregulated the expression of Merlin and neurofibromin in HSCs. The band intensities of the target proteins are expressed relative to those in the controls to obtain relative grayscale values (n=3). Compared with the controls the data are presented as the mean ± SD. **P* < 0.05; ***P* < 0.01.
