# Supplementary figures and images for: Loss of EPB41L3: a common molecular link in the tumorigenesis of neurofibromatosis types 1 and 2
Source: Front Oncol. 2026 May 20;16:1632602. doi: 10.3389/fonc.2026.1632602 (PMC13229769; doi:10.3389/fonc.2026.1632602)

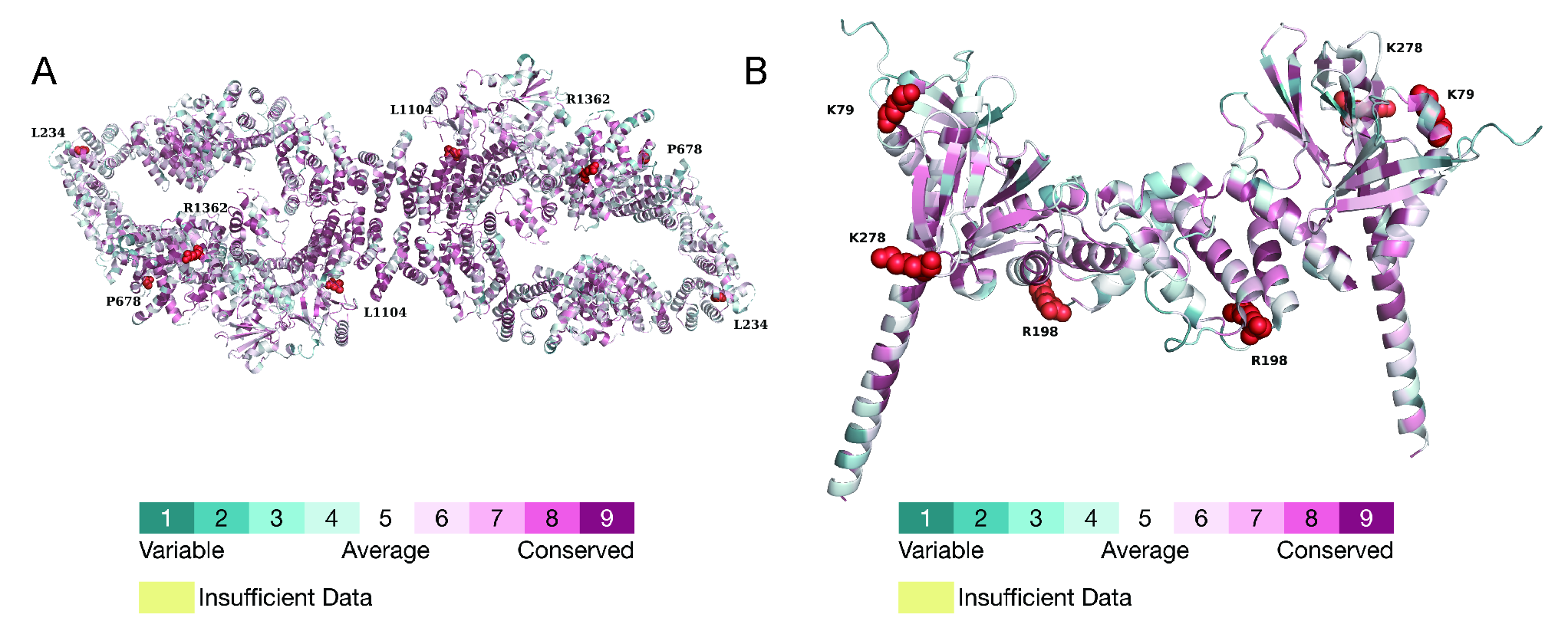

Supplement: Supplementary Figure 1 — Schematic diagram of proteins. (A) Consurf colored structure of the full-length human NF1 dimer. (B) Consurf colored structure of the full-length human NF2 dimer. [file Image1.tif]

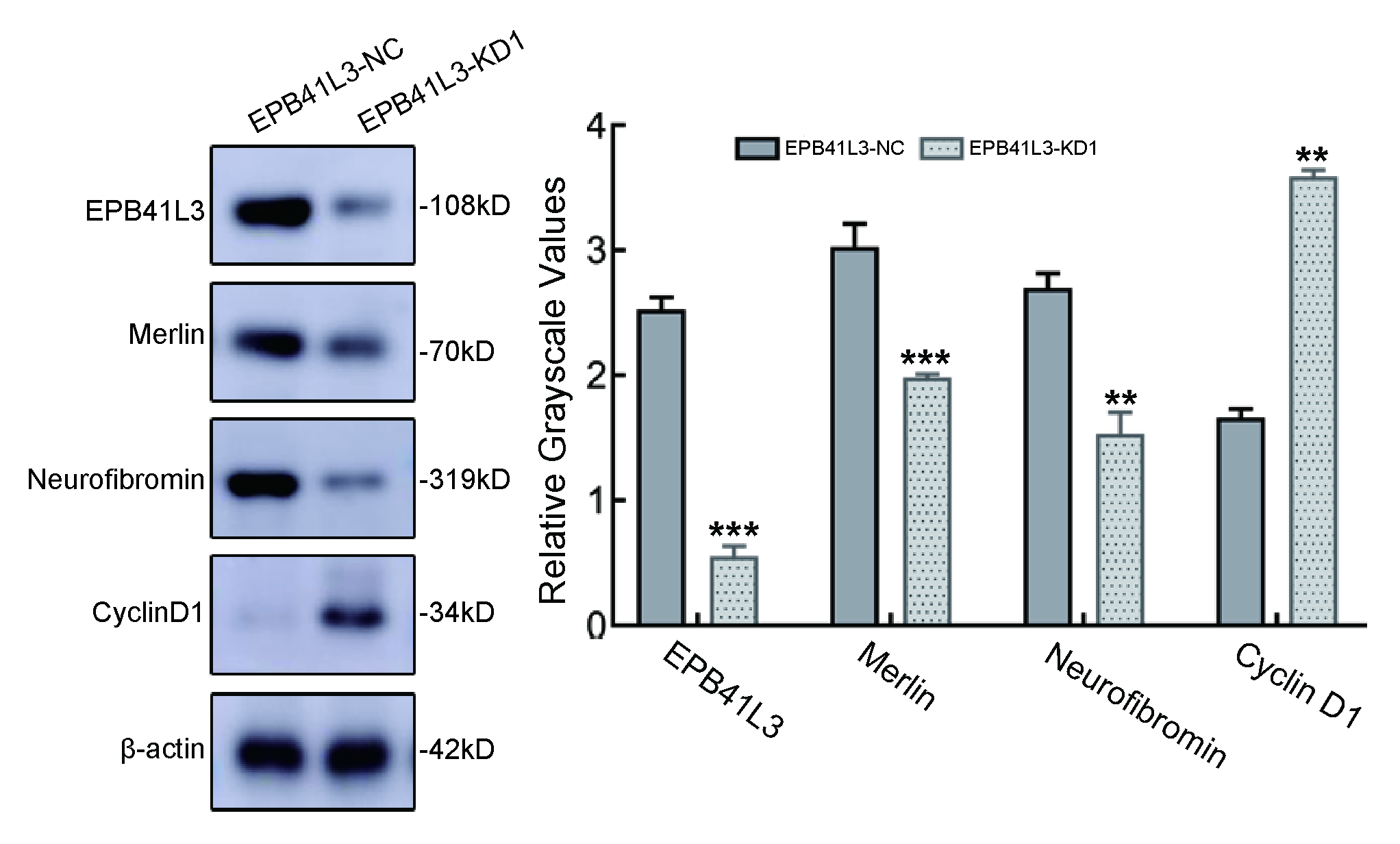

Supplement: Supplementary Figure 3 — EPB41L3 knockdown downregulated the expression of Merlin and neurofibromin in HSCs. The band intensities of the target proteins are expressed relative to those in the controls to obtain relative grayscale values (n=3). Compared with the controls the data are presented as the mean ± SD. *P < 0.05; **P < 0.01. [file Image3.tif]
